# Utility of Geriatric Syndrome Indicators for Predicting Subsequent Health Care Utilization in Older Adults in Taiwan

**DOI:** 10.3390/ijerph16030456

**Published:** 2019-02-04

**Authors:** Ching-Ju Chiu, Ya-Yun Cheng

**Affiliations:** 1 Institute of Gerontology, College of Medicine, National Cheng Kung University, No. 1, University Road, Tainan 70101, Taiwan; 2 School of Medicine, College of Medicine, National Cheng Kung University, No. 1, University Road, Tainan 70101, Taiwan; i54046322@mail.ncku.edu.tw

**Keywords:** geriatric syndromes, diabetes, dementia, depression, health care utilization

## Abstract

*Background*: The predictive utility of both individual and combined indicators of geriatric syndromes on subsequent emergency use and hospitalization is not clear. *Methods*: Nationally representative data on adults aged 65+ (N = 2345) (with 1148 male, 1197 female) in Taiwan were analyzed. The receiver operating characteristic (ROC) curve examined the diagnostic accuracy of the combined effects of geriatric syndromes on predicting health care utilization in three years. Negative binomial regressions identified the individual effect of each indicator with the control of sociodemographic and baseline health status. *Results*: The combined indicators of geriatric syndromes predicted future hospitalization of old-old (75+ yrs) diabetes patients, with area under the curve (AUC) = 0.709, 95% confidence interval (CI) = 0.635–0.782, and young-old patients (65–74 yrs) with mild cognitive impairment (AUC = 0.727, 95% CI = 0.610–0.845 for hospitalization and AUC = 0.770, 95% CI = 0.664–0.877 for emergency visits). As for individual indicators, while incontinence was the indicator having the most influence on hospitalization (incidence rate ratio (IRR) = 1.81, 95% CI = 1.21–2.72) and emergency visits (IRR = 1.78, 95% CI = 1.23–2.59) for general older adults (65+), and for old-old emergency visits, especially (IRR = 2.21, 95% CI = 1.39–3.49), falls was the most prominent indicator of hospitalization for young-old (65-74) adults (IRR = 1.61, 95% CI = 1.13–2.28). In addition, pain was another significant indicator for predicting future hospitalization of old-old diabetes patients (IRR = 1.61, 95% CI= 1.07–2.44). *Conclusions*: Combined indicators of geriatric syndromes effectively predict hospitalization in old-old (75+ yrs) diabetes patients and hospitalization and emergency visits in young-old (65–74 yrs) patients with cognitive impairment. Incontinence, falls, and pain were the most predictive independent geriatric assessment indicators.

## 1. Introduction

The emergence in old age of several complex health states that do not fall into discrete disease categories are commonly called geriatric syndromes. These complex health conditions are not exactly defined and generally include frailty, incontinence, falls, cognitive impairment, functional impairment, diabetes, depression, delirium, and pressure ulcers. Geriatric syndromes have been indicated to be a better predictor of death than assessing the presence or number of specific diseases [1] and therefore have become increasingly important assessment indicators in the area of geriatric medical care. 

In the past few years, the worldwide elderly population has grown rapidly. The number of published studies related to geriatric syndromes has also increased during the last decade [2]. It has been estimated that 44.9% of the population aged above 65 years in Taiwan has at least one geriatric syndrome symptom [3], indicating the common presence and importance of geriatric syndromes. Furthermore, researches have indicated that geriatric syndromes often co-occur with chronic diseases [4,5,6], and they are also associated with the risk of disability [5,7,8] and mortality [3,7,9,10]. Many studies have put a strong emphasis on the high correlation between combined geriatric syndromes and the use of medical resources, such as adverse hospitalization outcomes [11], number of hospitalizations [6,10,12], and hospital bed days [6,11]. All of the above-referenced studies have shown that geriatric syndrome indicators are highly important and are correlated with the health outcomes and health care utilization by the elderly, confirming that geriatric syndromes can be used as prognostic indicators. 

However, the above-referenced studies on geriatric syndromes have rarely distinguished the predictive utility of such indicators among the elderly in different age groups. While the number of elderly people increased from 1.89 million in 2000 to 3.19 million in 2017, the increase in the number of the old-old is even more significant [13]. According to the population data, even more importance is attached to health care for these much older elderly people. Older ages have also been pointed out to be the common risk factor for geriatric syndromes [3,14,15,16]. Moreover, studies have also indicated differences in health care utilization for younger and older elderly individuals. For example, studies have showed that an increase in age has a significant relationship with hospitalizations, fall injury hospitalizations, emergency visits, use of ambulances, and medical expenses [17,18,19]. In summary, the clinical differences between younger and older elderly individuals are noteworthy. However, according to the trend in current research, many studies have grouped people over the age of 65 into the same group for analysis, which is obviously contrary to the current situation.

In addition, it remains unclear whether the effects of geriatric syndromes on elderly people in different disease states can differ. In Taiwan, 86.3% of the elderly over the age of 65 self-reported that they had been diagnosed with a chronic disease by a physician. In 2013–2016, the prevalence of high blood sugar among Taiwanese people aged between 60–69, 70–79, and 80+ was 21.7%, 31.5, and 30.8%, respectively [20]. In addition, 19.6% adults aged 65 and over in Taiwan were shown to suffer from mild cognitive impairment [21], and the prevalence of depression based on the centers for epidemiological studies-depression (CESD) scale with cutoff points greater than 10 for Taiwanese adults aged 65–74, 75–84, and 85+ were 17.7%, 23.2%, and 20.6%, respectively [22]. It is worth exploring whether the predictive utilities of geriatric syndromes on subsequent emergency use and hospitalization differ for older adults with different chronic conditions.

If we look closely at the geriatric syndrome indicators individually, there have also been quite a few studies that suggest a relationship between different indicators of geriatric syndromes and hospitalization, emergency care, and nursing home care. For example, hospitalization has been significantly associated with falls [17,19], poor self-rated health [17], comorbidity [10], depression [17,23], dementia [23,24,25], and diabetes [26]. Also, emergency use has been shown to be significantly related to falls [17,18], poor self-rated health [17], depression [17], and dementia [25]. However, thus far, there is no research comparing the prognostic ability of each individual indicator in a study. Whether a single best indicator can be used for effective prognosis is an important topic worth exploring because it would simplify patient medical assessments.

Due to the lack of previous studies, this study intended to investigate the predictive utilities of both combined and individual indicators of eight geriatric syndromes (high blood sugar, depressive symptoms, cognitive impairment, physical disability, pain, incontinence, falls, and frailty) on subsequent health outcomes (number of emergency visits and hospitalizations) for older adults of different ages (65–74, 75+) with different chronic conditions (diabetes, mild cognitive impairment, and depressive symptoms).

## 2. Materials and Methods

### 2.1. Participants and Data Sources

This study drew data from older adults (65+) who participated in the 2011 Taiwan Longitudinal Study on Aging (TLSA) and agreed to have their data linked with the 2011–2014 National Health Insurance (NHI) claim data. The NHI was launched in 1995, has a coverage rate > 99% of the population and service contracts with 93% of the hospitals and clinics in Taiwan [27], and comprises a database of patients’ registration files and medical records. The data, including “ambulatory care expenditures by visit”, “inpatient expenditures by admission”, and “cause of death data”, were used in this study. The TLSA has included a national representative sample of adults aged 57 and above since 2003. Follow-up interviews were conducted in 2007 and 2011. Participants aged 65 and above in 2011 (N = 2426) were asked if they agreed to have their interview data linked with the National Health Insurance (NHI) Dataset, resulting in an analysis sample N = 2345, 96.67%.

### 2.2. Measures and Variables

#### 2.2.1. Geriatric Syndromes

This study assessed eight geriatric syndromes from 2011 data, including high blood sugar, depressive symptoms, cognitive impairment, physical disability, pain, incontinence, falls, and frailty. High blood sugar was measured with the question “Have you ever had high blood sugar?” Depressive symptoms were measured with the 10-item short form from the Centers for Epidemiological Studies-Depression (CES-D) scale [28]. The questionnaire began with the following instructions: “Please tell me how often you have felt this way during the past week”, and comprised 10 questions, including not feeling like eating, feeling that everything I did was an effort, restless sleep, feeling depressed, feeling lonely, people being unfriendly, feeling unable to “get going”, feeling sad, feeling happy, enjoying life. The scores ranged from 0 to 30, with higher scores representing higher levels of depressive symptoms. Using a dichotomy, participants scoring from 10 to 30 were defined as having depressive symptoms. [29] Cognitive impairment was measured with the 9-item Short Portable Mental Status Questionnaire (SPMSQ), with questions about orientation, memory, concentration, and mental tracking. [30,31] Defined based on the total number of correct answers out of nine questions, the scores ranged from 0 to 9, with higher scores indicating better cognitive function. Using a dichotomy, a score from 7–9 was defined as intact cognition, and a score < 7 was defined as mild cognitive impairment [31]. Physical disability was measured with the modified Katz Activities of Daily Living (ADL) scale, which evaluated six functions, including bathing, dressing, going to the toilet, transferring, continence, and feeding [32]. Difficulty in performing each ADL item was scored from 0 to 3: 0 for normal, 1 for slightly disturbed, 2 for severely disturbed, and 3 for functionally disrupted. This study modified the response score to range from 0 to 1: 0 for independent, 1 for dependent. The score was the total out of 6, with higher scores representing functionally disabled patients with worse activities of daily living. A dichotomy was used in this study, with a score ≧1 representing physically disabled. Pain was measured with the questionnaire about chronic diseases using the question, “Have you had pain in the last month? If yes, describe your pain as slight, moderate, or severe pain.” The total score ranged from 0 to 3: 0 for normal, 1 for slight, 2 for moderate, and 3 for severe. Using a dichotomy, a score ≧2 was defined as pain. Incontinence was identified using an NHI diagnosis (ICD-9: 788.3 and 625.6) and was defined as a patient had two outpatient visits or had one hospitalization in 2011. Falls was measured with the questionnaire about chronic diseases with the question, “How many times have you fallen in the past year?” Participants who had fallen two or more times were defined as having falls. Frailty was defined according to a previous study [33], using TLSA measures to define the symptoms of frailty. The symptoms included five criteria: shrinking or weight loss, weakness, exhaustion, slowness, and low physical activity. Each symptom was defined by the following questions or measurements: (1) Shrinking or weight loss was defined as having a poor appetite often (2–3 days) or most (4 days and above) in the last week; (2) weakness was defined as having difficulty carrying 12 kg of groceries; (3) exhaustion was defined as feeling that “I could not get going” or “I felt everything I did was an effort” often or most in the past week; (4) being slow was defined as having difficulty walking a distance of 200 to 300 meters; (5) low activity was defined using activities based on failure of the participant to garden, take a walk, jog, climb mountains, or engage in any other outdoor activities at least once or twice a week. The total score ranged from 0 to 5, with higher scores representing a higher level of frailty. Using a dichotomy, this study defined a score ≧3 as being frail [33].

#### 2.2.2. Prognostic Indicator

The number of emergency visits and number of hospitalizations in the National Health Insurance systems were separately recorded within the database of “medical emergency ambulatory care expenditures by visits”, and “inpatient expenditures by admissions” during the period from 2012 to 2014.

#### 2.2.3. Covariate Variables

The covariates were age, sex, comorbidity, self-rated health, death, and medical records. Comorbidity was defined with the questionnaire about chronic diseases in 2011, with the question “Have you ever had high blood pressure, diabetes, heart disease, stroke, cancers, lung disease, rheumatoid arthritis, or a hip fracture?” The score ranged from 0 to 8, with a higher score representing higher comorbidity. Self-rated health was measured with the question “Would you say that your health is excellent, very good, good, fair, or poor?” The score ranged from 1 to 5, with a higher individual score representing better self-rated health. Death was defined by the database for “the cause of death data”, noting whether the participant’s date of death was during the period from 2012 to 2014. Medical records (including number of emergency visits and hospitalizations) of participants in 2011 were analyzed with the NHI data, including ambulatory care expenditures by visits and inpatient expenditures by admissions. Three chronic disease conditions of patients were also explored in this study. One chronic disease condition was diabetes (N = 504), which was a self-reported diagnosis of diabetes in the TLSA. The second chronic disease condition was mild cognitive impairment (N = 320), which was defined by the SPMSQ questionnaire in the TLSA, for which the total score was 9. Participants were regarded as mildly cognitively impaired if they got scores < 7. The third chronic disease condition was depressive symptoms (N = 408). The patients used the CES-D questionnaire in the TLSA. Participants who scored 10 or more out of a total of 30 were considered to have depressive symptoms.

### 2.3. Statistical Analyses

All analyses were performed using SAS version 9.4 (SAS Institute, Cary, NC). First, the receiver operating characteristic (ROC) curve and the area under the ROC curve (AUC) were used to examine the diagnostic accuracy of the combined effects of geriatric syndromes on subsequent health outcomes. An AUC of 0.5 suggests no discrimination; 0.7 to 0.8 is considered acceptable, and greater than 0.8 is considered excellent. To identify the individual indicators of geriatric syndromes on subsequent health outcomes, a negative binomial regression model (NBR) was used, controlling for covariates of age, sex, number of comorbidities, self-rated health, follow-up status (death or not), and the baseline emergency room use and number of hospitalizations. The incidence rate ratio (IRR) presents the predictive utility while controlling for the covariates. 

### 2.4. Ethics Approval and Consent to Participate

This study was approved by the Institution Review Board (IRB) of National Cheng Kung University Hospital in Taiwan (No. B-ER-104-077).

## 3. Results

### 3.1. Descriptive Characteristics of the Participants 

Descriptive characteristics of the participants, including sample numbers, age, gender, self-reported health, number of emergency visits, number of hospitalization, comorbidities, and descendent, are provided, based on the presence or not of each of the geriatric syndrome indicators in 2011 (Table 1). Participants with cognitive impairment, physical disability, and frailty were older in age. Participants with any indicators of the geriatric syndromes, except for incontinence, had greater proportion of females, lower self-rated health, more comorbidities, and emergency and hospitalization visits at baseline. Differences in emergency visits and hospitalization between younger and older elderly people is shown (Table 2). The number of emergency visits and hospitalizations among the old-old adults (75+ yrs) were significantly higher compared to those of the young-old adults (65–74 yrs).

### 3.2. The Diagnostic Accuracy of the Combined Geriatric Syndrome Indicators for Predicting Subsequent Emergency Visits and Hospitalizations

The combined geriatric syndrome indicators in 2011 were significantly associated with 2012–2014 hospitalizations among older (75+ yrs) diabetes patients (AUC = 0.709, 95% CI = 0.635–0.782), and the emergency visits (AUC = 0.770, 95% CI = 0.664–0.877) or hospitalizations (AUC = 0.727, 95% CI = 0.610–0.845) among young-old adults (65–74 yrs) with mild cognitive impairment (Table 3). 

### 3.3. Effect of Individual Indicators of Geriatric Syndromes in Predicting the Number of Emergency Visits

The individual indicators of the 2011 geriatric syndromes in predicting the number of emergency visits from 2012 to 2014 after controlling for age, sex, comorbidities, self-rated health, and baseline health care utilization are shown (Table 4). Incontinence was the most prominent factor in predicting emergency visits in the three-year follow-up (IRR = 1.78, 95% CI = 1.23–2.59). The association was even more evident for old-old (75+ yrs) (IRR = 2.21, 95% CI = 1.39–3.49), or old-old adults (75+) with depressive symptoms (IRR = 3.01, 95% CI = 1.48–6.12). In addition, the pain indicator was also a critical factor for predicting subsequent emergency visits among diabetes patients aged 75 and older (IRR = 1.69, 95% CI = 1.11–2.57).

### 3.4. Effect of Individual Geriatric Syndrome Indicators in Predicting the Number of Hospitalizations

The individual indicators of the 2011 geriatric syndromes in predicting number of hospitalizations from 2012 to 2014 after controlling for age, sex, comorbidities, baseline self-rated health and health care utilization are shown (Table 5). In addition to the incontinence indicator, which was the most influential factor in predicting the number of hospitalizations (IRR = 1.81, 95% CI = 1.21–2.72), especially among participants aged 75 and over with depressive symptoms (IRR=3.33, 95% CI = 1.79–6.21) or mild cognitive impairment (IRR = 2.82, 95% CI = 1.27–6.25), it was found that pain and falls were also important indicators for predicting the number of hospitalizations. Pain was a crucial factor for predicting the hospitalization of old-old adults with diabetes (IRR = 1.61, 95% CI = 1.07–2.44), and falls was an especially prominent indicator for predicting hospitalizations in young-old adults (65–74 yrs) with mild cognitive impairment (IRR = 2.38, 95% CI = 1.29–4.42) or depressive symptoms (IRR = 2.70, 95% CI = 1.67–4.36).

## 4. Discussion

This is the first study comparing the predictive utilities of both individual and combined indictors of eight geriatric syndrome indicators on emergency care and hospitalization over a three-year period. It was found that combined indicators of geriatric syndromes are useful in predicting future health utilization among older (75+) diabetes patients, and young-old adults (65–74 years) with mild cognitive impairment. As for individual indicators, while incontinence was the most prominent item for predicting older adults’ future emergency and hospitalizations, especially in the case of old-old adults with depressive symptoms or cognitive impairment, falls was the most significant indicator for hospitalization of young-old adults, especially among young-old adults with mild cognitive impairment or depressive symptoms. In addition, pain was another significant indicator for predicting older (75+ yrs) diabetes patients’ future hospitalization and emergency visits. Combined indicators of geriatric syndromes were found to be predictive of hospitalization among older (75+) diabetes patients and emergency visits or hospitalizations among young-old adults (65–74 yrs) with mild cognitive impairment. Few past studies have mentioned how combined geriatric syndrome indicators affect patients with chronic conditions; instead, various studies have separately pointed out individual indicators of geriatric syndromes that may be predictive of elderly people’s health outcomes. For elderly people with diabetes, increase in age [34,35], and indicators of geriatric syndromes, including cognitive impairment and depression [36], are important predictors of hospitalization. As for elderly people with mild cognitive impairment, indicators such as increased age, functional status, diabetes, falls, and depression could worsen cognitive abilities and increase risk of mortality [37,38]. This study’s findings highlight the predictive ability of combined indicators of geriatric syndromes on future health utilization among elderly people with chronic conditions.

Therefore, in clinical care of the elderly, we call on patients with early-onset cognitive impairment and older diabetes patients, especially those older than 75, to undergo assessment of geriatric syndromes along with diagnosis of such chronic diseases. Furthermore, high-risk groups should take proactive preventive measures to prevent subsequent emergency visits and hospitalizations.

Incontinence was found in the present study to be the most influential factor predicting the future number of emergency visits and hospitalizations. Our findings echo previous research showing that incontinence is a common premorbid syndrome in acute care (ICU admission) [39,40]. Patients with incontinence are also shown to utilize significantly more medical resources, including the number of emergency visits, costs of emergency care, number of hospitalizations, and days of hospitalization [41]. Previous studies have also shown that increasing age is a consistently reported risk factor for incontinence [42,43], which may explain this study’s findings suggesting the predictive utilities of incontinence on older elderly people. However, patients’ embarrassment and unawareness of the need to report symptoms leads to a lack of diagnoses and possible interventions [40,42,44]. Thus, we call for more attention to and more proactive assessments of incontinence among the elderly under clinical care. 

This study also found the prominent role of falls in geriatric assessment, especially among younger (65–74 yrs) elderly or younger elderly people with mild cognitive impairment or depressive symptoms. In the past, many studies have mentioned the association between falls, hospitalization, and emergency visits. For example, a history of more than two falls and increased age have been shown to be important indicators of increased emergency visits and hospitalization within a year [17], and falls has been shown to be the main cause of emergency visits for the elderly [18,45]. Although this study only shows the prognoses of falls in younger elderly patients as it related to hospitalization, rather than the prognoses of falls as it is related to emergency visits and older elderly people, it still highlights the importance of fall prevention in the elderly, especially the younger elderly.

Pain was found to be a predictive indicator for hospitalization among the entire elderly population as well as emergency visits and hospitalizations among older patients (75+ yrs) with diabetes. Past studies have examined the effects of high rates of chronic pain and the severity of pain on overall activity and the other geriatric syndromes [46]. The findings from this study emphasize the importance of chronic pain in older adults and its effect on elderly care. As for patients with diabetes, past studies have indicated correlations between pain and the health outcomes of patients with diabetes, including increases in the frequency of visiting general practitioners [47], using home care services [47], and diabetes-related hospitalization in the past year [48]. Also, diabetic peripheral neuropathy pain (DPNP) is one of the most common complications in diabetic patients and is independently associated with higher age [49]. Meanwhile, past studies have also mentioned that patients with diabetes have higher health care utilization, such as rates of drug use [26] and hospitalization [26,50]. According to the findings of this study, the importance of pain in the prognosis of older diabetic patients should also be highlighted and is useful in efforts to decrease health care utilization.

The advantages of this study include the use of national data, so the sample size is large, highly representative, and heterogeneous. Second, this study uses a long-term tracking database, which is conducive to the confirmation of causalities and increased validity. Thirdly, according to self-evaluation, medical treatment information, and rich measurement data, where comorbidities, self-rated health, and basic hospitalizations are controlled, a relationship between geriatric syndromes, emergency visits, and hospitalization was observed. Furthermore, this study uses Eastern data to complement the shortcomings of most research using Western data. As for the limitations of this study, first of all, subject to the limitations of secondary data, the geriatric syndrome indicators only include 8 items (high blood sugar, depressive symptoms, cognitive impairment, physical disability, pain, incontinence, falls, and frailty). Indicators that have been common in past studies (such as sarcopenia, malnutrition, delirium, sensory defects, polypharmacy, pressure ulcers, etc.) were not explored in this study. Second, the geriatric syndromes and covariate variables estimated through self-reported questionnaires, such as high blood sugar, falls, comorbidities, or self-rated health, may be interfered with by the participants’ educational level, cognitive status or forgetfulness, becoming a bias of statistical analysis. In addition, this study does not discuss the cost or the difficulty and accuracy of testing through the use of indicators.

## 5. Conclusions

This study highlighted the importance of incontinence, falls, and pain in geriatric assessment since they showed the strongest correlation with subsequent health care utilization. The findings from this study shed light on the possibility that geriatric assessments may be simplified, thus decreasing medical costs.

## Figures and Tables

**Table 1:** Demographic and clinical characteristics of participants with different geriatric syndromes in 2011.

Characteristics	High Blood Sugar *N* = 2345			Depressive Symptoms *N* = 2002			Cognitive Impairment *N* = 1997			Physical Disability *N* = 2345		
	No	Yes	*p*-Value	No	Ye	*p*Value	No	Yes	*p*-Value	No	Yes	*p*-Value
*N*	1841	504		1594	408		1594	408		1812	533	
Age	77.0 ± 7.81	76.06 ± 7.32	0.0159	75.51 ± 7.39	76.56 ± 6.72	0.0060	75.51 ± 7.39	76.56 ± 6.72	0.0060	75.26 ± 7.20	82.02 ± 7.08	<0.0001
Age group			0.0557			0.0069			0.0069			<0.0001
65–74	796 (43.24)	242 (48.02)		807 (50.63)	176 (43.14)		807 (50.63)	176 (43.14)		942 (51.99)	96 (18.01)	
75+	1045 (56.76)	262 (51.98)		787 (49.37)	232 (56.86)		787 (49.37)	232 (56.86)		870 (48.01)	437 (81.99)	
Female	912 (49.54)	285 (56.55)	0.0053	731 (45.86)	271 (66.42)	<0.0001	731 (45.86)	271 (66.42)	<0.0001	883 (48.73)	314 (58.91)	<0.0001
Self-rated health	3.06 ± 0.98	2.80 ± 0.94	<0.0001	3.19 ± 0.93	2.33 ± 0.86	<0.0001	3.19 ± 0.93	2.33 ± 0.86	<0.0001	3.11 ± 0.95	2.39 ± 0.96	<0.0001
Number of emergency visits in 2011	0.5 ± 1.15	0.75 ± 2.11	0.0101	0.38 ± 0.9	0.77 ± 2.3	0.0009	0.38 ± 0.9	0.77 ± 2.3	0.0009	0.37 ± 0.9	1.16 ± 2.36	<0.0001
Number of hospitalizations in 2011	0.33 ± 0.91	0.54 ± 1.19	0.0005	0.24 ± 0.66	0.4 ± 0.89	0.0006	0.24 ± 0.66	0.4 ± 0.89	0.0006	0.22 ± 0.64	0.91 ± 1.57	<0.0001
Comorbidities	1.26 ± 1.08	2.71 ± 1.17	<0.0001	1.36 ± 1.13	1.97 ± 1.28	<0.0001	1.36 ± 1.13	1.97 ± 1.28	<0.0001	1.38 ± 1.14	2.23 ± 1.39	<0.0001
Descendent	308 (16.73)	117 (23.21)	0.0008	168 (10.54)	83 (20.34)	<0.0001	168 (10.54)	83 (20.34)	<0.0001	199 (10.98)	226 (42.40)	<0.0001
**Characteristics**	**Pain *N* = 2018**			**Incontinence *N* = 2345**			**Falls *N* = 2345**			**Frailty *N* = 2345**		
	**No**	**Yes**	***p*-Value**	**No**	**Yes**	***p*-Value**	**No**	**Yes**	***p*-Value**	**No**	**Yes**	***p*-Value**
*N*	1586	432		2296	49		2085	260		1706	639	
Age	75.76 ± 7.40	75.80 ± 6.81	0.9273	76.79 ± 7.72	76.88 ± 7.26	0.9399	76.53 ± 7.68	78.95 ± 7.70	<0.0001	75.23 ± 7.28	80.97 ± 7.28	<0.0001
Age group			0.3131			0.2835			<0.0001			<0.0001
65–74	785 (49.50)	202 (46.76)		1020 (44.43)	18 (36.73)		954 (45.76)	84 (32.31)		896 (52.52)	142 (22.22)	
75+	801 (50.50)	230 (53.24)		1276 (55.57)	31 (63.27)		1131 (54.24)	176 (67.69)		810 (47.48)	497 (77.78)	
Female	724 (45.65)	286 (66.20)	<0.0001	1170 (50.96)	27 (55.10)	0.5659	1041 (49.93)	156 (60.00)	<0.0001	790 (46.31)	407 (63.69)	<0.0001
Self-rated health	3.18 ± 0.93	2.37 ± 0.9	<0.0001	3.02 ± 0.98	2.72 ± 0.93	0.0509	3.07 ± 0.96	2.5 ± 0.96	<0.0001	3.19 ± 0.92	2.35 ± 0.91	<0.0001
Number of emergency visits in 2011	0.41 ± 1.31	0.69 ± 1.36	0.0001	0.54 ± 1.40	0.96 ± 1.90	0.1346	0.5 ± 1.39	0.96 ± 1.57	<.0001	0.4 ± 0.91	0.97 ± 2.22	<.0001
Number of hospitalization in 2011	0.25 ± 0.68	0.38 ± 0.86	0.0039	0.40 ± 1.16	0.65 ± 1.16	0.1321	0.36 ± 0.98	0.51 ± 0.98	0.0234	0.24 ± 0.68	0.74 ± 1.46	<.0001
Comorbidities	1.39 ± 1.14	1.84 ± 1.28	<0.0001	1.57 ± 1.25	1.86 ± 1.17	0.1083	1.51 ± 1.22	2.08 ± 1.36	<.0001	1.36 ± 1.13	2.15 ± 1.36	<.0001
Descendent	200 (12.61)	58 (13.43)	0.6527	417 (18.16)	8 (16.33)	0.7414	359 (17.22)	66 (25.38)	0.0013	199 (10.98)	226 (42.40)	<.0001

*Nte.* CI = Confidence interval. All values are *N* (%) or mean ± SD.

**Table 2:** Differences in emergency visits and hospitalization by age group.

	Entire Sample	Age Group		
Health Care Utilization	65+ *N* = 2345	65–74 *N* = 1038	75+ *N* = 1307	*p*-Value
Emergency visits at baseline, Mean ± SD	0.55 ± 1.42	0.37 ± 0.97	0.69 ± 1.68	<0.0001
Hospitalization at baseline, Mean ± SD	0.41 ± 1.16	0.27 ± 1.05	0.52 ± 1.23	<0.0001
Number of emergency visits at baseline, *N* (%)				<0.0001
0	1664 (70.96)	815 (78.52)	849 (64.96)	
1	411 (17.53)	145 (13.97)	266 (20.35)	
2+	270 (11.51)	78 (7.51)	192 (14.69)	
Number of hospitalization at baseline, *N* (%)				<0.0001
0	1819 (77.57)	866 (83.43)	953 (72.92)	
1	333 (14.20)	119 (11.46)	214 (16.37)	
2+	193 (8.23)	53 (5.11)	140 (10.71)	
Emergency visits during 2012–2014, Mean ± SD	1.91 ± 3.54	1.43 ± 3.65	2.29 ± 3.4	<0.0001
Hospitalization during 2012–2014, Mean ± SD	1.46 ± 2.63	1.16 ± 2.57	1.70 ± 2.66	<0.0001

**Table 3:** Area under curve (AUC) for the combined effects of geriatric syndrome indicators on older adults’ emergency visits and hospitalization, by age group and baseline health condition.

	Emergency Visits					
	65+		65–74		75+	
Participants	AUC	95% CI	AUC	95% CI	AUC	95% CI
Entire sample	0.597	0.573–0.621	0.615	0.582–0.648	0.570	0.534–0.606
With diabetes	0.654	0.603–0.704	0.618	0.553–0.683	0.670	0.582–0.757
With mild cognitive impairment	0.648	0.586–0.710	0.770	0.664–0.877	0.626	0.554–0.698
With depressive symptoms	0.571	0.514–0.629	0.647	0.567–0.728	0.617	0.540–0.695
	Hospitalization					
	65+		65–74		75+	
Participants	AUC	95% CI	AUC	95% CI	AUC	95% CI
Entire sample	0.615	0.591–0.639	0.603	0.568–0.637	0.603	0.569–0.636
With diabetes	0.676	0.625–0.723	0.586	0.516–0.656	0.709	0.635–0.782
With mild cognitive impairment	0.630	0.569–0.692	0.727	0.610–0.845	0.617	0.548–0.687
With depressive symptoms	0.638	0.584–0.692	0.692	0.615–0.769	0.643	0.571–0.714

*Note*. AUC = Area under curve, AUC ≥ 0.7 indicates satisfied diagnostic accuracy. CI = Confidence interval.

**Table 4:** Individual effects of geriatric syndrome indicators on number of emergency visits.

Geriatric Syndromes	Total Participants						Participants with Diabetes					
	Overall 65+ (*N* = 2345)		65–74 (*N* = 1038)		75+ (*N* = 1307)		Overall 65+ (*N* = 504)		65–74 (*N* = 242)		75+ (*N* = 262)	
	IRR (95% CI)	*p*-Value	IRR (95% CI)	*p*-Value	IRR (95% CI)	*p*-Value	IRR (95% CI)	*p*-Value	IRR (95% CI)	*p*-Value	IRR (95% CI)	*p*-Value
High blood sugar	1.03 (0.88–1.21)	0.7053	0.99 (0.78–1.26)	0.9370	1.09 (0.88–1.36)	0.4411	-	-	-	-	-	-
Depressive symptoms	1.04 (0.89–1.21)	0.6478	0.91 (0.71–1.18)	0.4881	1.07 (0.88–1.31)	0.4737	0.89 (0.66–1.20)	0.4451	0.69 (0.44–1.08)	0.1010	0.98 (0.64–1.48)	0.9159
Cognitive impairment	1.03 (0.88–1.22)	0.6887	1.17 (0.84–1.65)	0.3573	0.99 (0.82–1.19)	0.9006	1.18 (0.86–1.61)	0.3039	1.63 (0.96–2.76)	0.0715	1.00 (0.68–1.49)	0.9883
Physical disability	1.10 (0.93–1.31)	0.2724	1.22 (0.84–1.75)	0.2931	1.06 (0.87–1.30)	0.5348	1.13 (0.83–1.54)	0.4277	1.37 (0.80–2.33)	0.2541	1.02 (0.70–1.48)	0.9239
Pain	1.15 (0.99–1.34)	0.0714	1.12 (0.88–1.43)	0.3406	1.12 (0.92–1.37)	0.2613	1.28 (0.93–1.76)	0.1332	0.88 (0.53–1.46)	0.6125	1.69 (1.11–2.57)	0.0150
Incontinence	1.78 (1.23–2.59)	0.0024	1.04 (0.53–2.02)	0.9145	2.21 (1.39–3.49)	0.0007	1.32 (0.67–2.60)	0.4270	0.67 (0.14–3.33)	0.6264	1.58 (0.72–3.46)	0.2518
Fall	1.06 (0.88–1.28)	0.5592	1.29 (0.94–1.78)	0.1137	0.97 (0.77–1.22)	0.7842	1.31 (0.93–1.84)	0.1201	1.51 (0.92–2.50)	0.1048	1.15 (0.72–1.84)	0.5533
Frailty	0.93 (0.8–1.09)	0.3952	1.05 (0.78–1.41)	0.7560	0.87 (0.73–1.05)	0.1563	1.00 (0.74–1.35)	0.9998	1.00 (0.74–1.35)	0.9998	0.79 (0.54–1.15)	0.2098
	Participants with mild cognitive impairment						Participants with depressive symptoms					
	Overall 65+ (*N* = 320)		65–74 (*N* = 72)		75+ (*N* = 248)		Overall 65+ (*N* = 408)		65–74 (*N* = 176)		75+ (*N* = 232)	
	IRR (95% CI)	*p*-Value	IRR (95% CI)	*p*-Value	IRR (95% CI)	*p*-Value	IRR (95% CI)	*p*-Value	IRR (95% CI)	*p*-Value	IRR (95% CI)	*p*-Value
High blood sugar	1.29 (0.90–1.85)	0.1677	1.37 (0.74–2.52)	0.3132	1.23 (0.79–1.91)	0.3603	0.91 (0.66–1.25)	0.5628	0.77 (0.47–1.25)	0.2883	1.12 (0.74–1.71)	0.5825
Depressive symptoms	1.02 (0.75–1.40)	0.8778	1.08 (0.61–1.92)	0.7978	1.02 (0.71–1.46)	0.9318	-	-	-	-	-	-
Cognitive impairment	-	-	-	-	-	-	0.96 (0.71–1.30)	0.8100	1.50 (0.84–2.68)	0.1755	0.82 (0.58–1.15)	0.2497
Physical disability	0.99 (0.74–1.33)	0.9439	1.07 (0.57–2.04)	0.8259	0.98 (0.71–1.37)	0.9250	1.11 (0.84–1.47)	0.4642	1.14 (0.65–1.97)	0.6506	1.02 (0.73–1.40)	0.9246
Pain	0.98 (0.71–1.35)	0.8985	0.62 (0.35–1.11)	0.1099	1.10 (0.76–1.61)	0.6132	0.98 (0.75–1.29)	0.8947	1.13 (0.74–1.73)	0.5651	0.86 (0.62–1.21)	0.3991
Incontinence	1.72 (0.81–3.67)	0.1609	0.58 (0.09–3.81)	0.5726	1.97 (0.83–4.66)	0.1235	1.85 (1.03–3.32)	0.0384	0.29 (0.09–0.94)	0.0384	3.01 (1.48–6.12)	0.0024
Fall	1.03 (0.73–1.45)	0.8865	1.62 (0.92–2.85)	0.0955	0.89 (0.58–1.36)	0.5975	0.85 (0.63–1.16)	0.3037	1.03 (0.61–1.76)	0.9016	0.79 (0.55–1.13)	0.1925
Frailty	0.84 (0.62–1.14)	0.2637	0.85 (0.46–1.57)	0.6031	0.84 (0.59–1.19)	0.3315	0.86 (0.66–1.14)	0.2940	0.82 (0.53–1.26)	0.3583	0.81 (0.56–1.15)	0.2360

*Note*. IRR = Incidence rate ratio, CI = Confidence interval, All the coefficients adjusted for covariates of age, sex, number of comorbidities, self-rated health, follow-up status (descendent or not), and the baseline emergency room use and number of hospitalizations.

**Table 5:** Individual effects of geriatric syndrome indicators on number of hospitalizations.

Geriatric Syndromes	Total Participants						Participants with Diabetes					
	Overall 65+ (*N* = 2345)		65–74 (*N* = 1038)		75+ (*N* = 1307)		Overall 65+ (*N* = 504)		65–74 (*N* = 242)		75+ (*N* = 262)	
	IRR (95% CI)	*p*-Value	IRR (95% CI)	*p*-Value	IRR (95% CI)	*p*-Value	IRR (95% CI)	*p*-Value	IRR (95% CI)	*p*-Value	IRR (95% CI)	*p*-Value
High blood sugar	1.06 (0.89–1.27)	0.5319	1.14 (0.87–1.49)	0.3379	0.96 (0.76–1.22)	0.7476	-	-	-	-	-	-
Depressive symptoms	0.90 (0.76–1.07)	0.2420	0.93 (0.70–1.24)	0.6371	0.82 (0.66–1.02)	0.0760	1.07 (0.76–1.49)	0.7112	0.93 (0.53–1.63)	0.7939	0.97 (0.64–1.47)	0.8791
Cognitive impairment	1.04 (0.87–1.25)	0.6630	1.24 (0.85–1.81)	0.2678	1.01 (0.83–1.23)	0.9315	1.11 (0.77–1.59)	0.5888	1.38 (0.69–2.75)	0.3658	1.00 (0.67–1.49)	0.9940
Physical disability	1.14 (0.94–1.38)	0.1957	1.41 (0.95–2.11)	0.0888	1.05 (0.85–1.30)	0.6396	1.22 (0.86–1.75)	0.2662	1.15 (0.58–2.27)	0.6926	1.41 (0.96–2.07)	0.0793
Pain	1.23 (1.05–1.46)	0.0132	1.18 (0.91–1.54)	0.2129	1.16 (0.94–1.43)	0.1568	1.36 (0.96–1.94)	0.0857	0.96 (0.52–1.75)	0.8853	1.61 (1.07–2.44)	0.0238
Incontinence	1.81 (1.21–2.72)	0.0041	1.43 (0.71–2.87)	0.3185	1.97 (1.22–3.19)	0.0058	1.76 (0.82–3.80)	0.1471	1.16 (0.17–7.94)	0.8784	2.10 (1.00–4.39)	0.0493
Falls	1.11 (0.90–1.37)	0.3165	1.61 (1.13–2.28)	0.0080	0.93 (0.73–1.19)	0.5669	1.17 (0.79–1.75)	0.4365	1.18 (0.60–2.30)	0.6313	1.22 (0.78–1.93)	0.3814
Frailty	1.02 (0.86–1.22)	0.7873	1.29 (0.93–1.78)	0.1308	0.94 (0.77–1.14)	0.5255	1.06 (0.76–1.48)	0.7362	1.06 (0.76–1.48)	0.7362	0.98 (0.67–1.44)	0.9318
	Participants with mild cognitive impairment						Participants with depressive symptoms					
	Overall 65+ (*N* = 320)		65–74 (*N* = 72)		75+ (*N* = 248)		Overall 65+ (*N* = 408)		65–74 (*N* = 176)		75+ (*N* = 232)	
	IRR (95% CI)	*p*-Value	IRR (95% CI)	*p*-Value	IRR (95% CI)	*p*-Value	IRR (95% CI)	*p*-Value	IRR (95% CI)	*p*-Value	IRR (95% CI)	*p*-Value
High blood sugar	1.14 (0.79–1.64)	0.4812	1.48 (0.73–3.00)	0.2747	1.05 (0.69–1.61)	0.8231	1.52 (1.11–2.07)	0.0082	1.24 (0.76–2.02)	0.3865	1.48 (0.99–2.21)	0.0537
Depressive symptoms	0.91 (0.67–1.25)	0.5757	1.27 (0.66–2.43)	0.4769	0.81 (0.57–1.16)	0.2549	-	-	-	-	-	-
Cognitive impairment	-	-	-	-	-	-	1.09 (0.81–1.48)	0.5618	1.78 (0.98–3.23)	0.0597	0.93 (0.67–1.29)	0.6588
Physical disability	1.18 (0.87–1.60)	0.2757	1.50 (0.75–2.98)	0.2515	1.13 (0.81–1.59)	0.4657	1.28 (0.96–1.70)	0.0908	0.99 (0.56–1.75)	0.9776	1.30 (0.95–1.78)	0.0976
Pain	1.13 (0.82–1.55)	0.4689	1.42 (0.77–2.60)	0.2618	1.08 (0.74–1.58)	0.6952	1.23 (0.93–1.61)	0.1433	1.36 (0.87–2.12)	0.1746	1.10 (0.79–1.53)	0.5632
Incontinence	2.35 (1.14–4.82)	0.0203	0.48 (0.06–3.83)	0.4918	2.82 (1.27–6.25)	0.0107	2.59 (1.50–4.46)	0.0006	1.51 (0.59–3.87)	0.3950	3.33 (1.79–6.21)	0.0001
Falls	1.26 (0.89–1.79)	0.1906	2.38 (1.29–4.42)	0.0058	0.96 (0.62–1.48)	0.8544	1.29 (0.96–1.74)	0.0882	2.70 (1.67–4.36)	<0.0001	0.82 (0.58–1.17)	0.2706
Frailty	0.87 (0.64–1.19)	0.3882	1.09 (0.56–2.12)	0.7888	0.81 (0.58–1.15)	0.2475	1.24 (0.94–1.65)	0.1319	1.33 (0.85–2.08)	0.2077	1.10 (0.76–1.58)	0.6102

*Note*. IRR = Incidence rate ratio, CI = Confidence interval, All the coefficients adjusted for covariates of age, sex, number of comorbidities, self-rated health, follow-up status (descendent or not), and the baseline emergency room use and number of hospitalizations.
